# Sex-specific expression of CTNNB1 in the gonadal morphogenesis of the chicken

**DOI:** 10.1186/1477-7827-11-89

**Published:** 2013-09-11

**Authors:** Seung-Min Bae, Whasun Lim, Wooyoung Jeong, Jin-Young Lee, Jinyoung Kim, Fuller W Bazer, Gwonhwa Song

**Affiliations:** 1 Department of Agricultural Biotechnology, Seoul National University, Seoul 151-742, Republic of Korea; 2 Department of Animal Resources Science, Dankook University, Cheonan 330-714, Republic of Korea; 3 Center for Animal Biotechnology and Genomics and Department of Animal Science, Texas A&M University, College Station, Texas 77843-2471, USA; 4 Division of Biotechnology, College of Life Sciences and Biotechnology, Korea University, Seoul 136-713, Republic of Korea

**Keywords:** CTNNB1, Chicken, Gonad, Testis, Sertoli cell

## Abstract

**Background:**

Beta-catenin (CTNNB1), as a key transcriptional regulator in the WNT signal transduction cascade, plays a pivotal role in multiple biological functions such as embryonic development and homeostasis in adults. Although it has been suggested that CTNNB1 is required for gonad development and maintenance of ovarian function in mice, little is known about the expression and functional role of CTNNB1 in gonadal development and differentiation in the chicken reproductive system.

**Methods:**

To examine sex-specific, cell-specific and temporal expression of CTNNB1 mRNA and protein during gonadal development to maturation of reproductive organs, we collected left and right gonads apart from mesonephric kidney of chicken embryos on embryonic day (E) 6, E9, E14, E18, as well as testes, oviduct and ovaries from 12-week-old and adult chickens and performed quantitative PCR, *in situ* hybridization, and immunohistochemical analyses. In addition, localization of Sertoli cell markers such as anti-Müllerian hormone (AMH), estrogen receptor alpha (ESR1), cyclin D1 (CCND1) and N-cadherin (CDH2) during testicular development was evaluated.

**Results:**

Results of the present study showed that CTNNB1 mRNA and protein are expressed predominantly in the seminiferous cords on E6 to E14 in the male embryonic gonad, and are mainly localized to the medullary region of female embryonic gonads from E6 to E9. In addition, CTNNB1 mRNA and protein are abundant in the Sertoli cells in the testes and expressed predominantly in luminal epithelial cells of the oviduct, but not in the ovaries from 12-week-old and adult chickens. Concomitant with CTNNB1, AMH, ESR1, CCND1 and CDH2 were detected predominantly in the seminiferous cord of the medullary region of male gonads at E9 (after sex determination) and then maintained or decreased until hatching. Interestingly, AMH, ESR1, CCND1 and CDH2 were located in seminiferous tubules of the testes from 12-weeks-old chickens and ESR1, CCND1 and CDH2 were expressed predominantly in the Sertoli cells within seminiferous tubules of adult testes.

**Conclusions:**

Collectively, these results revealed that CTNNB1 is present in gonads of both sexes during embryonic development and it may play essential roles in differentiation of Sertoli cells during formation of seminiferous tubules during development of the testes.

## Background

Beta-catenin (CTNNB1), the vertebrate homolog of *Armadillo* in *Drosophila melanogaster*, plays important roles as a transcriptional co-activator in the WNT signal transduction cascade as well as linkage of the cadherin complex to the actin filament network during early embryogenesis and adult homeostasis [1]. In general, in the canonical WNT signaling pathway, cytoplasmic CTNNB1 is activated after binding of the WNT ligand to its transmembrane receptor Frizzled and its co-receptor, low-density lipoprotein receptor related protein 6. The activated CTNNB1 is shuttled into the nucleus to act as a transcriptional stimulator of the TCF/LEF family of DNA-bound transcription factors of the promoter regions of WNT target genes. Without WNT ligand, CTNNB1 is phosphorylated at serine residue 33 and 37 and provides a binding site for the E3 ubiquitin ligase, resulting in CTNNB1 ubiquitination and degradation by the destruction complex composed of Axin, *adenomatous polyposis coli* gene product (APC), glycogen synthase kinase 3 (GSK3), protein phosphatase 2A (PP2A), and casein kinase 1 (CK1) [2]. Thus, mutant CTNNB1 via mutation at the phosphorylation sites continuously activates WNT signaling cascades, leading to various diseases such as cancer [3]. For instance, it is well known that deregulation of the WNT/CTNNB1 signal transduction cascade results in colorectal carcinogenesis through excessive proliferation or renewal of stem cells resulting from a CTNNB1 mutation [4].

In the WNT signaling pathway, CTNNB1 also acts as a key player in a number of biological processes including development and differentiation of embryonic organs. For instance, during mouse embryonic development, *Ctnnb1*^−/−^ embryos fail to develop mesoderm and initiate gastrulation [5] and they have defects in anterior-posterior axis formation and fail to generate head structures [6]. On the other hand, CTNNB1 is involved in sex determination and differentiation in mice. Recently, Liu and colleagues reported that *Ctnnb1* is essential only for ovarian differentiation, but not necessary for testicular development in a conditional knockout mouse model (*Ctnnb1*^+/−^) [7]. However, little is known about the expression and function CTNNB1 in development of the chicken embryo, especially gonadal development, sex determination and differentiation. Indeed, in chickens, sex determination and differentiation is decided by a genetic ZZ/ZW system in which the male is homogametic (ZZ) and the female is heterogametic (ZW). In the chicken embryo (21-day embryonic period), the gonads on embryonic day (E) 3.5 exist in a bipotential undifferentiated state and are initially visible on the ventral surface of the embryonic kidneys [8]. The first signs of sex determination and differentiation occur at E6.5 when morphological changes include proliferation and thickening of the cortex of the left gonad as well as regression of the right gonad in the female. In contrast, the male embryo undergoes histological changes associated with formation of seminiferous cords in the medullary region and a reduction in size of the cortex without any morphological differences between the two gonads [9]. In mammals, the *sex determining region Y* (*SRY*), a well-known sex determining gene, *Sry*-*associated HMG*-*box gene 9* (*SOX9*) and *anti*-*Müllerian hormones* (*AMH*) are known molecular markers for fetal Sertoli cells of the differentiating embryonic testes [7]. Likewise, *double*-*sex and mab*-*3 related transcription factor 1* (*DMRT1*) is the avian Z-linked gene that acts as a vital factor for testis determination in chickens [10]. However, the sex-specific role of CTNNB1 in chicken gonadal development is unknown; therefore, we focused our investigation on that gene. Results of the present study indicate that CTNNB1 plays essential roles in development of the testes and, specifically, differentiation of Sertoli cells during formation of the seminiferous tubules.

## Methods

### Experimental animals and animal care

The experimental use of chickens for this study was approved by the Animal Care and Use Committee of Dankook University. White Leghorn (WL) chickens were exposed to a light regimen of 15 h light and 9 h dark with *ad libitum* access to feed and water, and subjected to standard poultry husbandry guidelines.

### Sex determination

Freshly laid eggs were incubated with intermittent rocking at 37°C under 60-70% relative humidity. Sex was determined on embryonic day E6.5. Approximately 0.2 ul of embryonic blood was collected from the dorsal aorta, diluted in 15 ul of 1× phosphate buffered saline (PBS, pH 7.4), and boiled at 95°C for 10 min to prepare the DNA template for PCR. Each 20 ul PCR reaction contained 2 ul of DNA template, 2 ul of PCR buffer, 1.6 ul of 2.5 mM dNTP mixture, 10 pmol of each forward and reverse primer of chicken W chromosome (F: 5’-CTA TGC CTA CCA CAT TCC TAT TTG C-3’ and R: 5’-AGC TGG ACT TCA GAC CAT CTT CT-3’), and 1 unit of Taq DNA polymerase. The thermal conditions for 35 cycles were 95°C for 30 sec, 66°C for 30 sec, and 72°C for 30 sec. Male or female sex was determined based on the strong bands detected for the W chromosome in the agarose gel after separation of PCR products by gel electrophoresis.

### Tissue samples

We collected left and right gonads apart from mesonephric kidney of chicken embryos at E6, E9, E14, E18, as well as testes, oviduct and ovaries from 12-week-old and adult chickens. After collecting gonads, tissue samples were stored at −80°C for extracting RNA or fixed in freshly prepared 4% paraformaldehyde in PBS (pH 7.4). After 24 h, embryos fixed in 4% paraformaldehyde were changed to 70% ethanol for 24 h and then dehydrated in a graded series of increasing concentrations of ethanol. Embryos were incubated in xylene for 3 h and embedded in Paraplast-Plus (Leica Microsystems, Wetzlar, Germany). Paraffin-embedded tissues were sectioned at 5 μm for further analyses.

### RNA isolation

Total cellular RNA was isolated from frozen tissues using Trizol reagent (Invitrogen, Carlsbad, CA) according to manufacturer’s recommendations. The quantity and quality of total RNA was determined by spectrometry and denaturing agarose gel electrophoresis, respectively.

### Quantitative RT-PCR analysis

Total RNA was extracted from each segment of oviduct at each time point using TRIzol (Invitrogen) and purified using an RNeasy Mini Kit (Qiagen). Complementary DNA was synthesized using a Superscript® III First-Strand Synthesis System (Invitrogen). Gene expression levels were measured using SYBR® Green (Biotium, Hayward, CA, USA) and a StepOnePlus™ Real-Time PCR System (Applied Biosystems, Foster City, CA, USA). The *glyceraldehydes 3*-*phosphate dehydrogenase* (*GAPDH*) gene was analyzed simultaneously as a control and used for normalization of data. *GAPDH* expression is assumed to be most stable among other house-keeping genes, so it is commonly used for normalizing for variations. Each target gene and *GAPDH* was analyzed in triplicate. Using the standard curve method, we determined expression of the examined genes using the standard curves and Ct values, and normalized them using *GAPDH* expression. The PCR conditions were 95°C for 3 min, followed by 40 cycles at 95°C for 20 sec, 60°C for 40 sec, and 72°C for 1 min using a melting curve program (increasing the temperature from 55°C to 95°C at 0.5°C per 10 sec) and continuous fluorescence measurement. ROX dye (Invitrogen) was used as a negative control for the fluorescence measurements. Sequence-specific products were identified by generating a melting curve in which the Ct value represented the cycle number at which a fluorescent signal was statistically greater than background, and relative gene expression was quantified using the 2^–ΔΔ^Ct method [11]. For the control, the relative quantification of gene expression was normalized to the Ct value for the control oviduct.

### *In situ* hybridization analysis

For hybridization probes, PCR products were generated from cDNA with the primers used for RT-PCR analysis. The products were extracted from the gel and cloned into pGEM-T vector (Promega, Madison, WI). After verification of the sequences, plasmids containing gene sequences were amplified with T7- and SP6-specific primers (T7:5’-TGT AAT ACG ACT CAC TAT AGG G-3’; SP6:5’-CTA TTT AGG TGA CAC TAT AGA AT-3’) then digoxigenin (DIG)-labeled RNA probes were transcribed using a DIG RNA labeling kit (Roche Applied Science, Indianapolis, IN). Tissues were collected and fixed in 4% paraformaldehyde, embedded in paraffin and sectioned at 5 μm on APES-treated (silanized) slides. The sections were then deparaffinized in xylene and rehydrated to diethylpyrocarbonate (DEPC)-treated water through a graded series of alcohol. The sections were treated with 1% Triton X-100 in PBS for 20 min and washed two times in DEPC-treated PBS. After washing in DEPC-treated PBS, the sections were digested with 5 μg/ml Proteinase K (Sigma-Aldrich, St. Louis, MO) in TE buffer (100 mM Tris–HCl, 50 mM EDTA, pH 8.0) at 37°C. After post-fixation in 4% paraformaldehyde, sections were incubated twice for 5 min each in DEPC-treated PBS and incubated in TEA buffer (0.1 M triethanolamine) containing 0.25% (v/v) acetic anhydride. The sections were incubated in a prehybridization mixture containing 50% formamide and 4× standard saline citrate (SSC) for at least 10 min at room temperature. After prehybridization, the sections were incubated overnight at 42°C in a humidified chamber in a hybridization mixture containing 40% formamide, 4× SSC, 10% dextran sulfate sodium salt, 10 mM DTT, 1 mg/ml yeast tRNA, 1 mg/ml salmon sperm DNA, 0.02% Ficoll, 0.02% polyvinylpyrrolidone, 0.2 mg/ml RNase-free bovine serum albumin and denatured DIG-labeled cRNA probe After hybridization, sections were washed for 15 min in 2× SSC at 37°C, 15 min in 1× SSC at 37°C, 30 min in NTE buffer (10 mM Tris, 500 mM NaCl and 1 mM EDTA) at 37°C and 30 min in 0.1× SSC at 37°C. After blocking with a 2% normal sheep serum (Santa Cruz Biotechnology, Inc., Santa Cruz, CA), the sections were incubated overnight with sheep anti-DIG antibody conjugated to alkaline phosphatase (Roche, Indianapolis, IN). The signal was visualized following exposure to a solution containing 0.4 mM 5-bromo-4-chloro-3-indolyl phosphate, 0.4 mM nitroblue tetrazolium, and 2 mM levamisole (Sigma Chemical Co., St. Louis, MO).

### Immunohistochemistry

Immunocytochemical localization of CTNNB1 protein was performed as described previously using a rabbit polyclonal antibody to CTNNB1 (catalog number #9582; Cell Signaling Technology) at a final dilution of 1:100. Negative controls included substitution of the primary antibody with purified non-immune rabbit IgG at the same final concentration.

### Statistical analyses

All quantitative data were subjected to analysis of variance (ANOVA) according to the general linear model (PROC-GLM) of the SAS program (SAS Institute, Cary, NC). All tests of significance were performed using the appropriate error terms according to the expectation of the mean square for error. Data are presented as mean ± SEM unless otherwise stated. Differences in the variances between E6 and E9 for each gonad were analyzed using the *F* test, and differences in the means were subjected to Student’s *t* test. Differences were considered significant at *P* < 0.05.

## Results

### Differential expression of CTNNB1 mRNA and protein during the male gonadal development and differentiation

**Figure 1:**
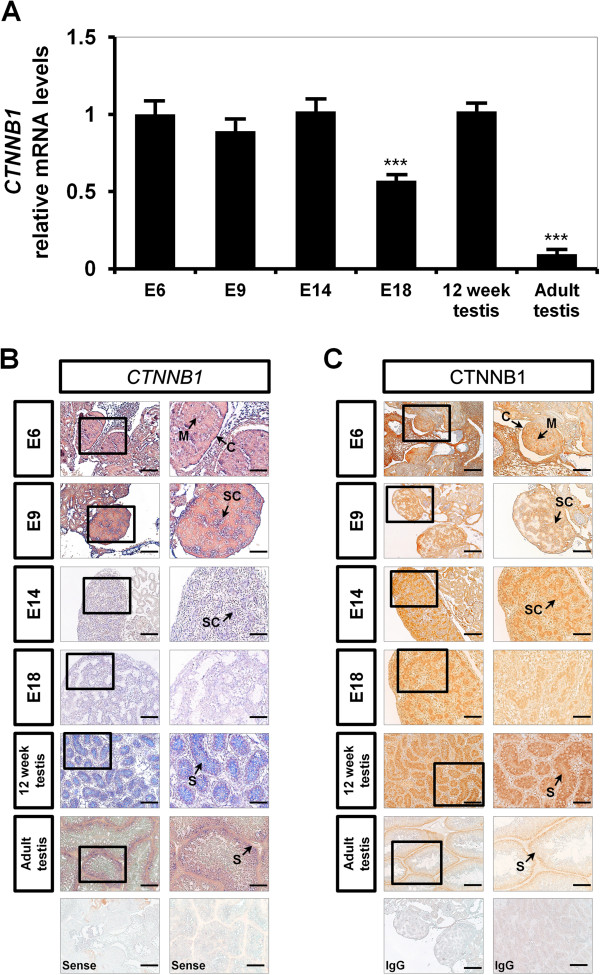
**Comparison of relative expression and localization of *CTNNB1 *mRNA and protein in male embryonic gonads during embryogenesis and reproductive organs from male chickens. [A]** Relative changes in *CTNNB1* mRNA expression were determined using quantitative RT-PCR. The asterisks denote statistically significant differences (***P < 0.001). **[B]** Cell-specific localization of *CTNNB1* mRNA was determined using *in situ* hybridization analysis. Cross sections of left and right gonads from E6 to E18 in male embryos and testes from 12-week-old and adult chickens were hybridized with antisense or sense cRNA probes. **[C]** Immunoreactive CTNNB1 protein during the development of chicken male gonad. For the IgG control, normal rabbit IgG was substituted for the primary antibody. Legend: *M* medullar region, *C* cortex region, *Sc* seminiferous cord, *S* Sertoli cells. *Scale bar* represents 100 μm. See Methods for a complete description of the methods.

To examine sex-specific, spatial- and temporal expression of CTNNB1 during gonadal development to maturation of reproductive organs, we collected gonads on embryonic day 6 (E6), E9, E14 and E18 from female and male embryos as well as testes, ovaries and oviducts from both sexes of chickens before and after sexual maturation. As illustrated in Figure 1A, in male gonads, *CTNNB1* mRNA expression was not different from E6 to E14, but its expression decreased around the time of hatching of the chick (E18). Interestingly, *CTNNB1* expression increased 1.4-fold (*P* < 0.001) in testes of immature chickens (12-weeks-old) and decreased (*P* < 0.001) in testes of adult males. *In situ* hybridization analysis showed that *CTNNB1* mRNA is expressed predominantly in the seminiferous cords of developing testes from E6 to E14 (Figure 1B). In testes from 12-week-old and adult male chickens, *CTNNB1* mRNA is abundant in the Sertoli cells. Consistent with these results, immunohistochemical analysis detected immunoreactive CTNNB1 protein mainly in the seminiferous cords of developing male gonads on E6 to E14 and it was also abundant in the Sertoli cells of testes from immature and adult male chickens (Figure 1C and Additional file 1: Figure S1).

### Differential expression of CTNNB1 mRNA and protein during the female gonadal development and differentiation

**Figure 2:**
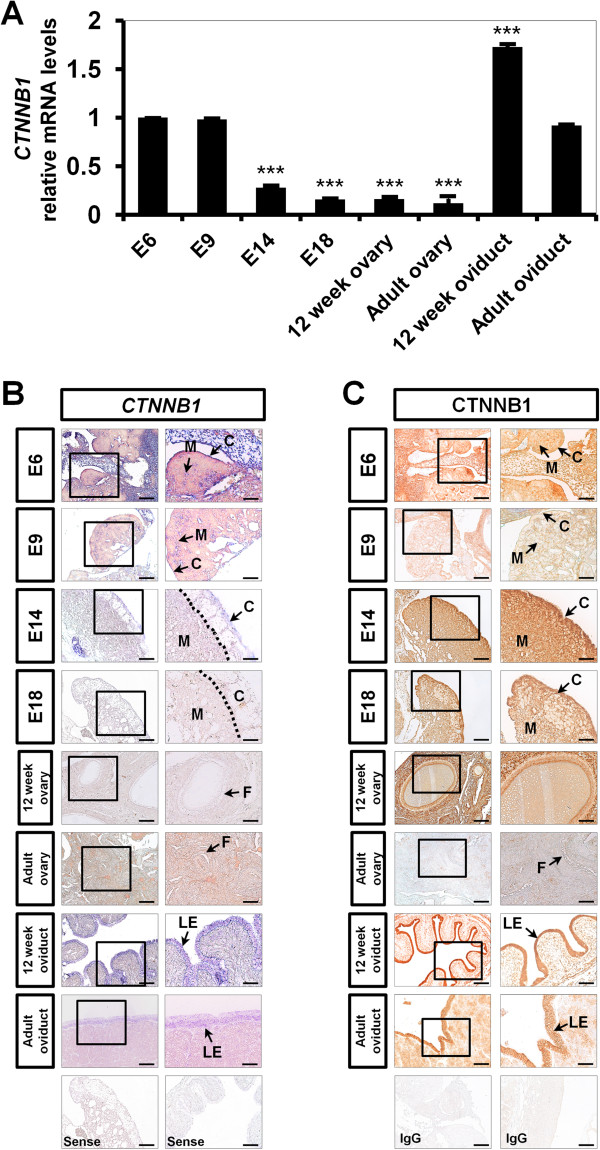
**Comparison of relative expression and localization of *CTNNB1 *mRNA and protein in female embryonic gonads during embryogenesis and reproductive organs from female chickens. [A]** Relative changes in *CTNNB1* mRNA expression were determined using quantitative RT-PCR. The asterisks denote statistically significant differences (***P < 0.001). **[B]** Cell-specific localization of *CTNNB1* mRNA was determined using *in situ* hybridization analysis. Cross sections of left gonad from E6 to E18 in female embryos, oviduct and ovary from 12-week-old and adult chickens were hybridized with antisense or sense cRNA probes. **[C]** Immunoreactive CTNNB1 protein during the development of chicken female gonad. For the IgG control, normal rabbit IgG was substituted for the primary antibody. Legend: *M* medullar region, *C* cortex region, *Sc* seminiferous cord, *S* Sertoli cells, *LE* luminal epithelium. *Scale bar* represents 100 μm. See Methods for a complete description of the methods.

Although the gonads of chick embryos have no left-right asymmetrical morphology in either sex as for mammalian species before sex determination, the onset of sex-specific differentiation on E6.5 drives histologically apparent differences in gonadal development that is asymmetric. Thus, most female birds have a functional left ovary and oviduct whereas the right gonad degenerates. Therefore, we focused our study on the left gonad from female embryos. As shown in Figure 2A, the expression of *CTNNB1* mRNA decreased steadily from E9 to E18 (*P* < 0.001). However, *CTNNB1* mRNA then increased about 11-fold (*P* < 0.001) in the oviducts from 12-weeks-old chicken as compared to that on E18 and about 6-fold (*P* < 0.001) in adult oviducts, whereas there in no significant difference of *CTNNB1* mRNA in ovaries from immature and mature chickens. *In situ* hybridization analysis showed that *CTNNB1* mRNA is mainly localized in the medullary region of embryonic gonads from E6 to E9 and weakly detected in the cortex region of gonads from E14 to E18. In the chicken oviduct, *CTNNB1* mRNA is predominantly expressed in luminal epithelial cells, but not in any other cell-types. However, little or no expression of *CTNNB1* mRNA was detected in the ovaries from immature and mature chickens. In support of these results, immunohistochemistry demonstrated that immunoreactive CTNNB1 protein was localized in medullary and cortical regions of embryonic gonads from E6 to E9 and from E14 to E18, respectively. Of particular note, CTNNB1 protein was abundant in the luminal epithelium of the chicken oviduct.

### Expression of sertoli cell development-related genes, AMH, ESR1, CCND1 and CDH2 during testicular development

**Figure 3:**
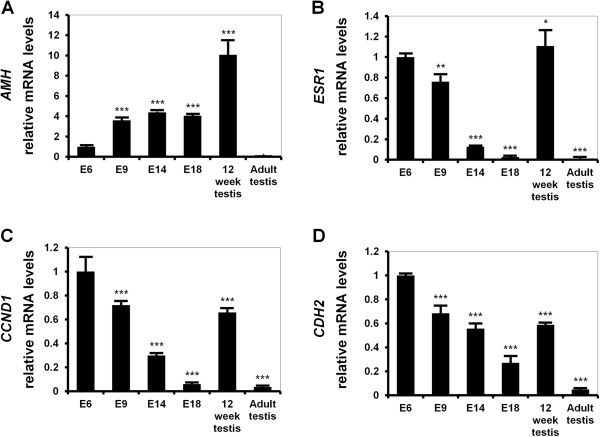
**Comparison of relative expression of *AMH*, *ESR1*, *CCND1 *and *CDH2 *mRNAs in male embryonic gonads during embryogenesis and testes from male chickens.** Relative changes in expression of **[A]***AMH*, **[B]***ESR1*, **[C]***CCND1* and **[D]***CDH2* mRNAs were determined using quantitative RT-PCR. The asterisks denote statistically significant differences (***P < 0.001, **P < 0.01 and *P < 0.05). Legend: *E* embryonic day. See Methods for a complete description of the methods.

Recently, we reported that CTNNB1 plays important roles in development of the chicken oviduct. Thus, in the present study, we focused on the expression and function of CTNNB1 in the development of Sertoli cells in gonads of roosters. Indeed, anti-Müllerian hormone (AMH) is known as a Sertoli cell marker because it is secreted by Sertoli cells for regression of the Müllerian duct in female embryos [12]. In addition, estrogen mediates physiological effects in male testes via its cognate receptor estrogen receptor alpha (ESR1) [13-15] and cyclin D1 (CCND1) plays a key role in integrin-mediated adhesion in formation of tight junctions between Sertoli cells [16]. N-Cadherin as a well-known adhesion protein was expressed by immature Sertoli cells in response to estrogen [17]. Therefore, we performed quantitative PCR analyses to determine quantitative changes in expression of *CTNNB1*, *AMH*, *ESR1*, *CCND1* and *CDH2* mRNAs during the course of testicular development. As illustrated in Figure 3, *AMH* mRNA increased 3.6-fold (*P* < 0.001) at E9 and was maintained until hatching. However, it increased 10-fold (*P* < 0.001) in the testes from 12-weeks-old chickens as compared to E6. On the other hand, expression of *ESR1*, *CCND1* and *CDH2* mRNAs decreased gradually until E18 and then dramatically increased about 42-, 11- and 2.5-fold (*P* < 0.001), respectively, in testes from 12 week old males as compared to expression levels at E18.

### Localization of sertoli cell development-related genes, AMH, ESR1, CCND1 and CDH2 during testis development

**Figure 4:**
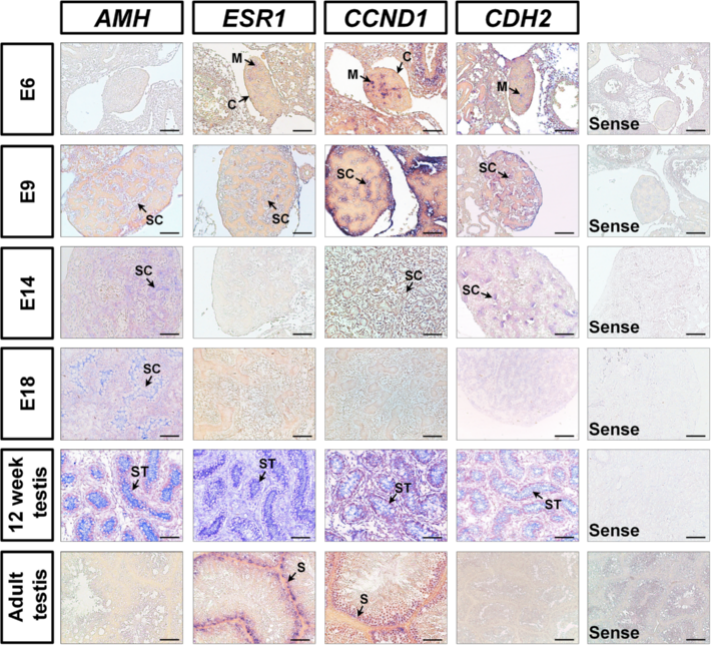
***In situ *hybridization analyses of *AMH*, *ESR1*, *CCND1 *and *CDH2 *mRNAs in male embryonic gonads during embryogenesis and testes from male chickens.** Cell-specific localization of *AMH*, *ESR1*, *CCND1* and *CDH2* mRNAs was determined using *in situ* hybridization analysis. Cross-sections of right and left gonads from E6 to E18 male embryos, and testes from 12-week-old and adult chickens were hybridized with antisense or sense cRNA probes. Legend: *M* medullar region, *C* cortex region, *Sc* seminiferous cord, *ST* seminiferous tubules, *S* Sertoli cells. *Scale bar* represents 100 μm. See Methods for a complete description of the methods.

To determine the localization of *AMH*, *ESR1* and *CCND1* mRNAs in the developing gonads and testes from chickens, we performed *in situ* hybridization analyses (Figure 4). The expression of *AMH* mRNA is evident in the seminiferous cord at E9 (after sex determination) and maintained until hatching. *AMH* mRNA was then localized to seminiferous tubules in the testes from 12-weeks-old males during development and differentiation of Sertoli cells within the seminiferous tubules, but this transcript was not detected in seminiferous tubules of adult male chickens. *ESR1* mRNA was also expressed in seminiferous cords at E9 and then its expression decreased to E18. However, *ESR1* mRNA was abundant in Sertoli cells of the seminiferous tubules of testes from 12-weeks-old male chickens. *CCND1* and *CDH2* mRNAs were expressed in seminiferous cords of the medullary region of male gonads from E6 to E9 and in the seminiferous tubules of testes from 12 week old males. Interestingly, *CCND1* was expressed predominantly in the nuclei of Sertoli cells within the seminiferous tubules of testes from adult male chickens.

## Discussion

Results of the present study provide comprehensive information on expression of CTNNB1 during embryogenesis and in the maturation of male and female gonads of chickens. Our results also indicate stage of development and cell-specific patterns of expression of AMH, ESR1 and CCND1 during embryonic development, as well as during post-hatching sexual maturation of roosters that are very similar to that for CTNNB1 in testes of the rooster. These results support our hypothesis that CTNNB1 plays important roles in differentiation of Sertoli cells and in the formation of seminiferous tubules during testes development.

CTNNB1, a member of the armadillo family of proteins, has the classical armadillo repeat domain and the C-terminal domain. Interestingly, the N-terminal region (known as a Helix C) of the C-terminal domain connects with the end of the armadillo repeat domain resulting in formation of a CTNNB1 superhelical core that is necessary to recruit and bind various CTNNB1 co-activators for increasing levels of transactivation in the WNT signal transduction cascades [18]. In addition, the sequence of CTNNB1 is highly conserved among various species. For instance, chicken CTNNB1 amino acid sequence shares 99-, 99- and 85% identity with mouse, human and *Xenopus*, respectively [19]. In the present study, we assessed sex-specific, cell-specific and temporal expression of CTNNB1 in gonads on selected embryonic days from female and male embryos. In male gonads, CTNNB1 mRNA and protein were expressed predominantly in the seminiferous cords of developing testes between E6 and E14 (Figure 1). But, in female gonads, expression of CTNNB1 mRNA and protein were mainly localized to the medullary region of embryonic gonads from E6 to E9 and weakly detected in the cortex region of gonads from E14 to E18 (Figure 2). In chickens, undifferentiated gonads consist of an outer cortex and underlying medulla, and most mesenchymal cells are localized in the medullary region and most primordial germ cells are in the cortical region [20]. However, during morphological differentiation after onset of sex determination, medullary cords transform into seminiferous cords in male embryonic gonads and the cortex is diminished in developing testes [20]. Interestingly, CTNNB1 mRNA and protein were abundant in Sertoli cells of testes from immature and mature male chickens (Figure 1). In the chicken oviduct, CTNNB1 mRNA and protein were predominantly detected in luminal epithelial cells, but not in any other cell-type (Figure 2). However, little or no expression of *CTNNB1* mRNA was detected in ovaries from either immature or mature female chickens. Therefore, CTNNB1 likely has an essential role in testis development and Sertoli cell differentiation, but it is not required for development and differentiation of the ovary in female chickens.

Morphological and functional aspects of the reproductive organs (i.e. two gonads, and Müllerian and Wolffian ducts) of mammalian male and female embryos are initially indistinguishable. But, with initiation of sexual differentiation occurs in response to testicular hormones such as testosterone and anti-Müllerian hormone (AMH), also known as Müllerian inhibiting substance [21]. AMH, a glycoprotein in the transforming growth factor-beta family, is secreted by Sertoli cells and transduces its signal via transmembrane receptors such as AMH type II receptor, after the onset of testis determination in the male fetus [22]. As a result, there is regression of the Müllerian duct and testosterone produced by Leydig cells, promotes Wolffian duct development [23]. AMH induces accumulation of CTNNB1 in the cytoplasmic and nuclear compartments of peri-Müllerian mesenchymal cells which likely alters target gene expression patterns in the cells leading to regression of the Müllerian ductal epithelial cells [24]. This result indicates that CTNNB1 might be a critical regulator in the AMH signaling pathway [23]. Thus, we examined the expression AMH in gonads of male chicken embryos in this study and demonstrated that it was expressed predominantly in seminiferous cords from E9 to hatching (Figures 3 and 4). In addition, *AMH* mRNA was expressed in seminiferous tubules in testes from 12-weeks-old chickens during development and differentiation of Sertoli cells in seminiferous tubules, but not in Sertoli cells in testes of sexually mature male chickens. These results support the idea that AMH initially produced by the Sertoli cells of the embryonic testes in the fetal male remain elevated levels during childhood, but decreases to low levels from puberty to mature adult male stage in life [25]. Therefore, results of our current study suggest that AMH-activated CTNNB1 plays an important role in sex determination, differentiation and testes development in male chickens.

In the male reproductive system, androgens from Leydig cells are metabolized to estrogens by the P450 aromatase enzyme localized mainly to Sertolic cells and germ cells and estrogen is involved in various biological and physiological functions of testes [26]. In general, estrogen mediates various physiological effects via specific estrogen receptors, particularly estrogen receptor alpha (ESR1) and –beta (ESR2) that accompany activation of SRC and several signaling cascades, such as the MAPK and EGFR cell signaling pathways. Hence, this activation of estrogen-dependent signal transduction likely contributes to the proliferation of Sertoli cells and interactions between Sertoli cells and germ cells in mice. For instance, Esr1 knockout mice have impaired spermatogenesis with reduced sperm numbers and abnormal sperm function leading to infertility [27,28]. In addition, *Esr1*^−/−^ and *Esr1*^−/−^/*Esr2*^−/−^ mice exhibit depletion of germ cells in the seminiferous tubules and defective structural changes such as a marked dilation of straight tubules and rete testis [29]. These results indicate that estrogen and its receptor are essential for normal spermatogenesis, development of seminiferous tubules and fertility in males. In the present study, ESR1 was expressed predominantly in seminiferous cords at E9 and then its expression decreased until after hatching. ESR1 was abundant in seminiferous tubules of testes of male chickens at 12 weeks of age and expressed specifically in Sertoli cells of adult testes; changes are coordinate with abundant expression of CTNNB1. Indeed, there are many reports that estrogen induces WNT/CTNNB1 signaling pathways in various organs and disease states. For example, when estrogen levels increase or decrease, there is coordinate accumulation or reductions in amounts of activated CTNNB1 proteins in the nucleus during the proliferative and secretory phases of the menstrual cycle in women [30]. Moreover, administration of exogenous estrogen to mice increased nuclear localization of Ctnnb1 in epithelial cells of the mouse uterus [31]. Therefore, our current results suggest that estrogen-ESR1 signaling via CTNNB1 is likely to be involved in development and differentiation of seminiferous tubules and Sertoli cells in male chickens.

In the Sertoli cells, estrogen also regulates expression of cyclin D1 (CCND1), as a well-known D-type cell cycle regulator, through SRC/MAPK pathways [32], that plays a pivotal roles in the G_1_/S phase during spermatogonial cells proliferation [33,34]. Indeed, CCND1 is one of the essential factors in cell adhesion. In the testis, integrins mediate formation of Sertoli cell tight junction complexes and several studies suggested that CCND1 plays a key role in integrin-mediated cell adhesion [16]. In addition, expression of CCND1 is stimulated by focal adhesion kinase in response to cell adhesion signals [35] and expression is directly increased by integrin-liked kinase which is a required for cell adhesion and interactions with integrin beta subunits [36]. Interestingly, over-expression of CTNNB1 increased CCND1 protein levels and consequently induced rapid progression of the cell cycle [37]. On the other hand, in the non-canonical WNT pathway, CTNNB1 and N-cadherin participate in cell-cell adhesion [38]. Of particular note, N-cadherin can mediate adhesion between Sertoli cells and spermatogenic cells and also serve as a marker of Sertoli cells. Moreover, CTNNB1 has an important function to regulate adhesion and signaling pathways at Sertoli cell-germ cell junctions [39-41]. In the present study, CCND1 was specifically and abundantly expressed in the seminiferous cords of male gonads between E6 and E9 and in seminiferous tubules of testes at 12 weeks of age. Interestingly, CCND1 was predominantly expressed in Sertoli cells within the seminiferous tubules of adult testes. Therefore, based on previous reports and our present results, we suggest that CCND1 expression increases coordinately with increases in expression of CTNNB1 to play important roles in increasing proliferation of seminiferous epithelial cells and Sertoli cells within the seminiferous tubules.

## Conclusions

Collectively, results of the present study provide the first molecular and cellular evidence for distinct sex-specific, cell-specific and temporal expression of CTNNB1 during gonadal development from embryonic stages to maturation of the reproductive organs, specifically the gonads, in chickens. Further, our results revealed coordinate changes in expression of CTNNB1, along with AMH, ESR1 and CCND1 during development of the seminiferous tubules and differentiation of Sertoli cells in testes of male chickens. Therefore, results of the present study provide new insights into sex-specific expression of CTNNB1 during sex determination and differentiation as well as during testicular development during post-hatching stages of development in male and female chickens.

## Competing interests

The authors declare that they have no competing interests.

## Authors’ contributions

SB and WL performed all the experiments and wrote the manuscript. WJ, JL and JK contributed to the tissue sampling. FWB participated in the design of the study, data analysis and drafted the manuscript. GS, as corresponding author, designed the experiments, analyzed experimental data and drafted the manuscript. All authors read and approved the final manuscript.

## Supplementary Material

Additional file 1: Figure S1 ESR1 protein was localized to the medullary region of embryonic gonads from E6 to E18 and decreased in abundance in that region between E6 and E18. Sertoli cells in testes on 12 week of embryo had abundant amounts of ESR1 whereas ESR1 protein was less abundant in adult testes. As shown in Figure 1C and Supplementary Figure 1, the cell specific expression of *ESR1* mRNA was coincident with localization of the CTNNB1 protein, which suggests that CTNNB1 is closely related to the development of Sertoli cells in an ESR1-dependent manner. Click here for file
