# Supplementary figures and images for: Sex-specific expression of CTNNB1 in the gonadal morphogenesis of the chicken
Source: Reprod Biol Endocrinol. 2013 Sep 11;11:89. doi: 10.1186/1477-7827-11-89 (PMC3847165; doi:10.1186/1477-7827-11-89)

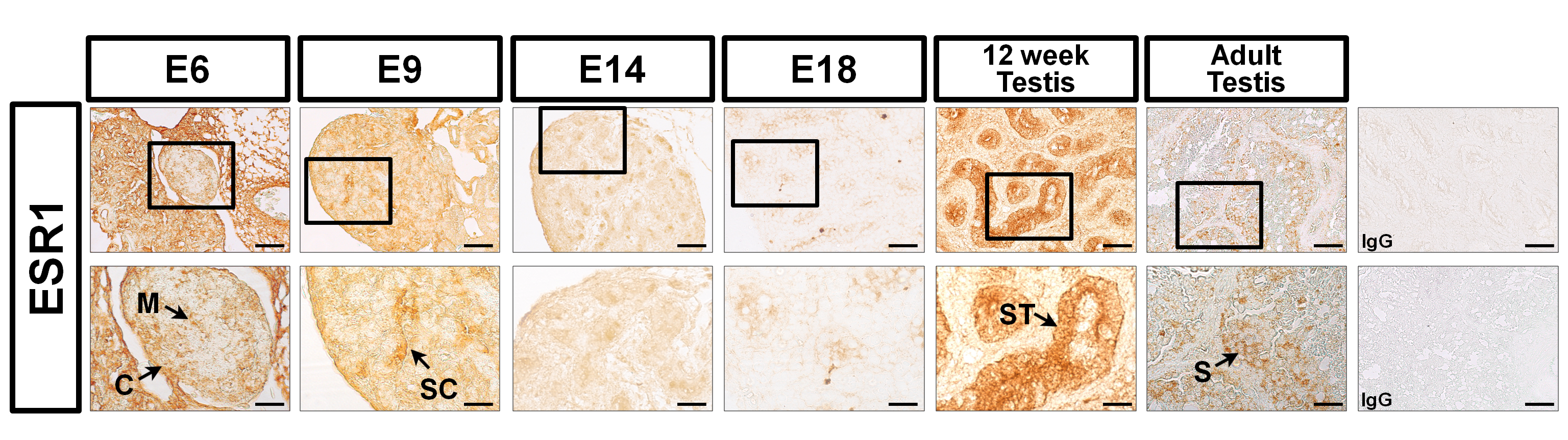

Supplement: Additional file 1: Figure S1 — ESR1 protein was localized to the medullary region of embryonic gonads from E6 to E18 and decreased in abundance in that region between E6 and E18. Sertoli cells in testes on 12 week of embryo had abundant amounts of ESR1 whereas ESR1 protein was less abundant in adult testes. As shown in Figure 1C and Supplementary Figure 1, the cell specific expression of ESR1 mRNA was coincident with localization of the CTNNB1 protein, which suggests that CTNNB1 is closely related to the development of Sertoli cells in an ESR1-dependent manner. [file 1477-7827-11-89-S1.jpeg]
